# Effect of dietary coenzyme Q10 supplementation on the growth rate, carcass characters and cost effectiveness of broiler fed with three energy levels

**DOI:** 10.1186/2193-1801-3-518

**Published:** 2014-09-11

**Authors:** Marappan Gopi, Manika Ragavan Purushothaman, Dooraisamy Chandrasekaran

**Affiliations:** Department of Animal Nutrition, Veterinary College and Research Institute, Namakkal, India

**Keywords:** Body weight gain, Coenzyme Q10 (CoQ10), Cost effectiveness, Energy levels, Feed efficiency

## Abstract

The objective of this experiment was to study the effect of dietary supplementation of Coenzyme Q10 on broiler growth rate, carcass characteristics and cost of production. A biological trial was carried out with 270 broiler chicks fed with coenzyme Q10 at 0, 20 and 40 mg/kg of diet at each of the three energy levels. At the end of 42 days growth period the birds were sacrificed and the samples were analysed. Feed intake was comparable in all the energy and CoQ10 combinations, but higher body weight gain and better feed efficiency with less feed cost per kilogram weight gain was observed in high energy group supplemented with 20 mg of CoQ10/kg diet. The dressing percentages, weight of giblet, liver, spleen, abdominal fat, intestinal length were not significantly altered by CoQ10 supplementation. The heart weight, gizzard weight and ascites heart weight (AHI) were significantly decreased due to CoQ10 supplementation. Hence, birds fed with high energy diet supplemented with 20 mg CoQ10 per kg of diet had higher production performance.

## Introduction

The growth rate of broilers has increased over the decades as a result of continuous selective breeding by the broiler breeders (Schmidt et al. 2009). Feeding and management systems for this level of production had to be developed concurrently to exploit the full genetic potential. To achieve this, the poultry nutritionist attempt to increase the density of the nutrients in the diet. This leads to increased oxidative stress on the broilers and depresses the growth performance (Shi-bin et al. 2007). Oxidative stress occurs when there is an imbalance between the production of oxygen radicals and antioxidants in the body. Reactive oxygen species (ROS) are mainly mitochondrial derived and directly affects the vascular remodeling and also causes pulmonary hypertension and growth rate (Bautista-Ortega et al. 2010). Broilers fed to an energy dense diet were more susceptible to oxidative stress (Cardoso et al. 2010), whereas restricted feeding decreases oxidative damage (Ozkan et al. 2010). However, an early feed restriction has severely affected the growth performance and lipid metabolism in broilers (Zhan et al. 2007; Saber, et al. 2011). To overcome this oxidative stress due to nutrient dense diets, few dietary additives like anti-stress - vitamins, minerals, antioxidants (lycopene, lutein), herbals like *Withania somnifera* (aswagantha), *Ocimum tenuiflorum* (tulsi), etc. have been tried with varying degree of success. Hence, there is a need for use of compounds which might protect the birds from oxidative stress and with better nutrient utilization efficiency. One such unique compound is Coenzyme Q10 (CoQ10) which involved in mitochondrial oxidative phosphorylation and also acts as an antioxidant.

Coenzyme Q10, a naturally occurring lipophilic antioxidant, present in the mitochondria of all body cells. Being a lipid-soluble compound, it is involved in the mitochondrial adenosine triphosphate (ATP) production - bioenergetics (Geng et al. 2004a). Geng *et al.* (2007) observed a significantly higher body weight gain in broilers when CoQ10 was supplemented at 40 mg/kg of diet with comparable feed intake and feed efficiency. Huang *et al.* (2011) supplemented CoQ10 at 20 and 40 mg/kg of broiler diet and had significantly better body weight gain upto 21 days of age. Nakamura *et al.* (1996) got an increased survival rate and low ascites mortality when he supplemented CoQ9 at 0.2 per cent in broiler diet than the non-supplemented group and the body weight at 56 days and feed efficiency were not significant. CoQ10 in its reduced form acts as a free radical scavenger (Forstmark–Andre et al. 1997) and preferred over α-tocopherol (Tang et al. 2001). Its major function in the blood is as an antioxidant (Alleva et al. 1997). CoQ10 is involved in the prevention of lipid peroxidation and also in regeneration of the other endogenous antioxidants (Navas et al. 2007). Serum or liver malonaldehyde (MDA) a product of lipid peroxidation was significantly decreased by CoQ10 supplementation (Geng et al. 2004a), indicative lipid peroxidation prevention. The activity of superoxide dismutase (SOD) was found to increase in CoQ10 supplementation at 40 mg/kg in broiler diet (Geng et al. 2004b; Huang et al. 2011) and in rats (Lakomkin et al. 2005) and anti-ROS capacity in broilers was increased by CoQ10 supplementation (Geng et al. 2004b). In broilers, only few studies using CoQ10 have been conducted with the objective of reducing ascites mortality in temperate regions. This study was carried out under prevailing local conditions where the cost of energy is very high and heat stress is chronic to know whether supplementation of CoQ10 could able to meet both these factors.

## Materials and methods

### Birds and diets

All procedures in the experiment were in carried out in accordance with the Institutional Animal Ethics Committee and conformed to the “Guidelines for the Care and Use of Animals in Research”. The experiment was carried out during the month of October to December. The daily maximum and minimum temperature and relative humidity inside the experimental shed during the experimental period was recorded and temperature humidity index was calculated as per Tao and Xin (2003)


where, THI = temperature humidity index, °C, T_db_ = dry-bulb temperature, °C, T_wb_ = wet-bulb temperature, °C. The mean maximum and minimum temperatures, relative humidity and temperature humidity index (THI) are presented in Table 1. The temperature, relative humidity and THI inside the experimental house during the trial period ranged from 19.69 - 32.31°C, 44.08 - 76.29 per cent and 24.46 - 31.07°C respectively. The experiment was carried out with 270 day - old Cobb 400 broiler chicks which consist of nine treatments with three replicates each containing ten chicks. Feed grade coenzyme Q10 (Qzyme®, manufactured by Agranco, USA Code No. DV100-12) was obtained and its activity was estimated based on the method described by Vicas et al. (2009) against standard CoQ10 obtained from Sigma. The CoQ10 preparation used in this biological trial was quantified for CoQ10 and it was found to be 205 units or 205 mg per gram. The treatment includes three levels of CoQ10 namely 0, 20 and 40 mg/kg of diet each at three energy levels namely normal energy (NE) as specified by the breeder, low energy – LE (normal energy minus 100 kcal/kg of diet) and high energy - HE (normal energy plus 100 kcal/kg of diet). The treatments were labelled as NE without CoQ10, LE without CoQ10, HE without CoQ10 and 20 and 40 mg of CoQ10 supplemented in the above three levels of energy diet. All diets contain same levels of calorie: protein, calorie: lysine and calorie: methionine ratio. The ingredients and nutrient compositions were shown in the Table 2.

**Table 1:** **Temperature, relative humidity and temperature humidity index inside the experimental house**

Period	Temperature (°C)		Relative humidity (%)		Morning (°C)		Evening (°C)		Temperature-Humidity Index THI (°C)	
	Max.	Min.	Max.	Min.	Dry bulb	Wet bulb	Dry bulb	Wet bulb	Morning	Evening
0-2 weeks	29.46	22.21	76.29	61.57	25.86	23.21	29.43	24.07	25.46	28.63
3-4 weeks	31.80	20.67	71.47	48.07	25.64	21.93	31.43	23.21	25.08	30.20
5-6 weeks	32.31	19.69	74.00	44.08	24.93	21.80	32.33	23.93	24.46	31.07

**Table 2:** **Ingredients and nutrients composition (% DM) of different broiler diets**

Ingredients (%)	0-2 Weeks			3-4 Weeks			5-6 Weeks		
	NE	LE	HE	NE	LE	HE	NE	LE	HE
Maize	54.2	58.96	49.88	53.9	60.4	50.5	57.22	62.0	52.79
DORB	0.07	0.13	0.035	0.36	0.081	1.10	0.00	0.00	0.89
SOYA	39.58	36.58	41.78	37.5	32.3	37.9	32.58	29.99	34.26
Rice bran Oil	2.12	0.00	4.30	4.24	2.13	6.30	5.86	3.61	8.00
Calcite	1.54	1.83	1.53	1.52	1.50	1.50	1.73	1.77	1.48
DCP	0.90	0.92	0.88	0.95	1.01	0.94	1.10	1.12	1.05
Salt	0.180	0.163	0.181	0.181	0.164	0.182	0.184	0.166	0.184
Lysine	0.298	0.319	0.302	0.150	0.220	0.189	0.167	0.187	0.168
Methionine	0.304	0.293	0.319	0.283	0.282	0.315	0.272	0.257	0.293
Threonine	0.00	0.035	0.035	0.00	0.00	0.00	0.018	0.028	0.018
**Nutrients (%)**									
Crude protein	22.65	21.80	23.30	21.65	20.35	21.75	19.70	19.00	20.21
Metabolisable Energy (Kcal/kg)*	3000	2900	3100	3125	3025	3225	3250	3150	3350
Calcium	0.96	0.96	0.96	0.95	0.95	0.95	0.90	0.90	0.90
Available phosphorus	0.45	0.45	0.45	0.47	0.47	0.47	0.46	0.46	0.46
Lysine*	1.42	1.37	1.47	1.25	1.21	1.29	1.14	1.10	1.18
Methionine*	0.62	0.60	0.64	0.59	0.57	0.61	0.55	0.53	0.57

Mineral mixture addition at 0.1% supplied (in mg) manganese-91, zinc - 91, iron-85, iodine- 1.82, copper −30.24 and cobalt – 0.365. Vitamin premix added at 0.5 g per kg feed supplied vitamin A-16500 IU, B_2_-13 mg, D_3_-3200 IU and vitamin K-2 mg, thiamine −5 mg, pyridoxine −8 mg, Niacin −320 mg, cyanocobalamine −0.05 mg, vitamin E −95 mg, calcium D pantothenate −27.5 mg and folic acid −14 mg, calcium −30.1 mg. Coccidiostat added at 0.5 g per kg feed supplied 125 mg of Di-nitro-ortho-toluamide. Antibiotic (10% Oxy tetracycline) 0.5 g was added per kg of feed.

*Calculated values.

### Production parameters

The birds were reared under deep litter system and maintained under standard managemental practices for a period of 42 days. The individual body weight and replicate feed intake were recorded at day one, weekly intervals and body weight gain and feed efficiency were worked out. At the end of 42 days of growth period, nine male birds per treatment (three birds per replicate) were randomly selected sacrificed. The pre-slaughter, dressed, giblet, spleen, heart and abdominal fat weights and intestinal length were recorded. The right and left ventricles were separated and weighed. The ascites heart index was calculated using Huchzermeyer and Ruyck (1986) equation method.


Production score was used as an index in the production of broiler chickens (Suzuki and Shibata 1989). It was calculated as follows:


The economics of raising broilers upto six weeks with different levels of CoQ10 supplementation and nutrient density was calculated based on the actual cost of feed per kg weight gain.

### Statistical analyses

The data collected on various parameters were analysed by one way ANOVA method as per the method of Snedecor and Cochran (1989) and the means of different experimental groups were tested for statistical significance (P < 0.05) by Duncan’s multiple range test (Duncan 1955).

## Results

### Production performance

The effect of CoQ10 levels and energy levels on the body weight gain, feed intake, feed efficiency and production score at biweekly intervals and overall (0 to 6 weeks) growth period are presented in Tables 3, 4 and 5 respectively.

**Table 3:** **Influence of Coenzyme Q10 and energy each at three levels on body weight gain (g) and production score at different growth phases**

Treatments	0-2 weeks	3-4 weeks	5-6 weeks	0-6 weeks	Production Score
NE	258^abc^ ± 07	672 ± 23	1007^a^ ± 32	1948^a^ ± 42	276.8^abc^ ± 11.5
LE	250^ab^ ± 06	632 ± 24	1023^a^ ± 32	1904^a^ ± 49	258.9^a^ ± 21.3
HE	260^abc^ ± 08	688 ± 23	1122^bc^ ± 35	2062^bc^ ± 47	322.2^cd^ ± 8.8
NE + 20	243^a^ ± 07	653 ± 28	980^a^ ± 46	1932^ab^ ± 53	270.2^ab^ ± 27.2
LE + 20	253^ab^ ± 08	629 ± 24	1030^ab^ ± 28	1912^a^ ± 47	268.2^ab^ ± 15.6
HE + 20	291^d^ ± 08	702 ± 15	1136^c^ ± 41	2129^c^ ± 49	357.1^d^ ± 3.4
NE + 40	251^ab^ ± 08	654 ± 25	969^a^ ± 38	1910^a^ ± 47	264.4^a^ ± 16.8
LE + 40	265^bc^ ± 08	595 ± 26	1039^abc^ ±35	1900^a^ ± 42	268.8^ab^ ± 0.6
HE + 40	275^cd^ ± 09	644 ± 24	1068^abc^ ± 36	1987^ab^ ± 55	314.2^bcd^ ± 4.7
P value	0.0005	0.07	0.009	0.005	0.001

Means with atleast one common superscript in a column do not differ significantly (P > 0.05).

**Table 4:** **Influence of Coenzyme Q10 and energy each at three levels on feed intake (g) at different growth phases**

Treatments	0-2 weeks	3-4 weeks	5-6 weeks	0-6 weeks
NE	312 ± 24	970 ± 18	1930^a^ ± 80	3212 ± 72
LE	322 ± 34	940 ± 59	2184^d^ ± 86	3446 ± 06
HE	304 ± 09	874 ± 15	1931^a^ ± 41	3217 ± 45
NE + 20	287 ± 25	908 ± 31	1949^a^ ± 35	3144 ± 87
LE + 20	308 ± 21	897 ± 60	2134^cd^ ± 14	3339 ± 82
HE + 20	310 ± 09	853 ± 19	1931^a^ ± 72	3094 ± 73
NE + 40	304 ± 18	916 ± 40	2013^abc^ ± 58	3233 ± 52
LE + 40	322 ± 05	840 ± 30	2117^bcd^ ± 23	3279 ± 26
HE + 40	301 ± 12	793 ± 28	1974^abc^ ± 22	3068 ± 61
P value	0.95	0.09	0.02	0.09

Means with atleast one common superscript in a column do not differ significantly (P > 0.05).

**Table 5:** **Influence of Coenzyme Q10 and energy each at three levels on feed efficiency of broilers at different growth phases**

Treatments	0-2 weeks	3-4 weeks	5-6 weeks	0-6 weeks
NE	1.220 ± 0.073	1.445 ± 0.047	1.921^abcd^ ± .111	1.706^bcd^ ± 0.044
LE	1.308 ± 0.179	1.489 ± 0.096	2.145^d^ ± 0.146	1.814^d^ ± 0.116
HE	1.152 ± 0.032	1.273 ± 0.036	1.811^ab^ ± 0.018	1.561^abc^ ± 0.019
NE + 20	1.164 ± 0.109	1.405 ± 0.127	2.006^bcd^ ±0.139	1.749^cd^ ± 0.094
LE + 20	1.283 ± 0.179	1.438 ± 0.144	2.073^bcd^ ± .032	1.749^cd^ ± 0.071
HE + 20	1.054 ± 0.021	1.215 ± 0.023	1.698^a^ ± 0.026	1.453^a^ ± 0.024
NE + 40	1.250 ± 0.123	1.405 ± 0.096	2.085^cd^ ± 0.126	1.755^d^ ± 0.078
LE + 40	1.229 ± 0.043	1.412 ± 0.010	2.037^bcd^ ± .020	1.726^bcd^ ± 0.005
HE + 40	1.041 ± 0.042	1.231 ± 0.024	1.849^abc^ ± .043	1.544^ab^ ± 0.012
P value	0.45	0.22	0.04	0.01

Means with atleast one common superscript in a column do not differ significantly (P > 0.05).

In the 0–2 week’s growth phase, the feeding of CoQ10 at 20 mg/kg in high energy diet had significantly higher body weight gain than the unsupplemented groups and normal and low energy birds fed with 20 or 40 mg/kg of CoQ10. During the 3–4 week’s growth phase, there was no significant difference in body weight gain due to the CoQ10 supplementation at each energy level. However, in the 5–6 week’s growth phase and 0–6 week’s growth period supplementation of CoQ10 at either of the levels did not improve the body weight gain over the respective unsupplemented groups. The body weight gain (0–6 weeks) was significantly higher in high energy diet supplemented with 20 mg of CoQ10/kg (2129 g) when compared to the other energy (1900 to 1987 g) and CoQ10 levels except for high energy unsupplemented group (2062 g).

During the entire growth period there was no effect of CoQ10 at different energy levels on feed intake and feed efficiency. In the 5–6 weeks growth phase, the supplementation at both the levels of CoQ10 (20 and 40 mg/kg) did not alter the feed intake over the unsupplemented group. However, supplementation of CoQ10 in low energy diet at both the levels (20 and 40 mg/kg) reduced the feed intake and it was comparable to the normal and high energy diets. In the finisher phase, supplementation at both the levels of CoQ10 did not alter the feed efficiency over the respective unsupplemented groups. The production score was not significantly affected by CoQ10 supplementation over the respective unsupplemented energy groups. Numerically the feed efficiency and production score was best in high energy diet supplemented with CoQ10 at 20 mg/kg.

### Slaughter parameters and ascites heart index

The results of slaughter parameters – dressed weight, giblet weight, liver, spleen, gizzard weight, per cent of abdominal body fat and ascites heart index are presented in Table 6.

**Table 6:** **Influence of Coenzyme Q10 and energy each at three levels on slaughter parameters**

Treatments	Dressing Percentage (%)	Giblet weight (g)	Gizzard weight (g)	Liver weight (g)	Heart weight (g/kg body weight)	Abdominal Fat (% of body weight)	Spleen weight (g)	Intestinal Length (cm)	Intestine length (cm/kg body weight)	Ascites Heart Index (AHI) (RV/TV)
NE	70.0 ± 0.8	112.6 ± 7.0	42.3 ± 2.0	59.7 ± 5.2	10.6^ab^ ± 0.4	1.03^bc^ ± 0.10	3.2 ± 0.4	226.1 ± 5.1	112.2^c^ ± 2.4	0.255^ab^ ± 0.003
LE	70.1 ± 0.6	98.0 ± 2.9	40.7 ± 1.7	46.1 ± 2.2	11.2^bc^ ± 0.6	1.11^bc^ ± 0.10	2.9 ± 0.4	211.8 ± 6.8	101.9^ab^ ± 4.0	0.261^bc^ ± 0.005
HE	70.3 ± 0.7	103.2 ± 4.8	39.1 ± 1.0	51.6 ± 3.6	12.6^c^ ± 0.8	1.33^d^ ± 0.22	2.9 ± 0.3	223.2 ± 5.1	101.1^ab^ ± 3.5	0.273^c^ ± 0.007
NE + 20	70.1 ± 0.9	105.2 ± 1.7	37.7 ± 1.4	56.7 ± 1.5	10.9^bc^ ± 0.6	0.90^b^ ± 0.07	3.8 ± 0.4	222.9 ± 4.8	108.0^ab^ ± 2.8	0.258^bc^ ± 0.005
LE + 20	70.0 ± 0.9	101.7 ± 2.5	38.6 ± 1.2	52.2 ± 1.9	10.9^bc^ ± 0.8	1.08^bc^ ± 0.13	3.7 ± 0.5	213.2 ± 7.1	103.0^ab^ ± 3.5	0.258^bc^ ± 0.007
HE + 20	70.4 ± 0.6	103.9 ± 1.8	37.7 ± 1.1	55.6 ± 2.5	10.7^ab^ ± 0.8	1.21^cd^ ± 0.14	2.6 ± 0.4	216.8 ± 3.8	98.0^a^ ± 1.7	0.256^ab^ ± 0.007
NE + 40	70.1 ± 0.8	101.3 ± 3.3	37.4 ± 1.6	54.2 ± 2.5	9.7^ab^ ± 0.6	0.68^a^ ± 0.09	3.8 ± 0.5	216.5 ± 4.7	106.7^bc^ ± 3.1	0.248^ab^ ± 0.005
LE + 40	70.2 ± 0.8	97.0 ± 4.8	35.7 ± 1.3	52.4 ± 3.9	8.9^a^ ± 0.7	1.07^bc^ ± 0.15	3.1 ± 0.4	223.1 ± 4.3	109.4^bc^ ± 3.5	0.241^a^ ± 0.006
HE + 40	70.1 ± 0.6	101.7 ± 4.1	38.9 ± 1.3	51.7 ± 3.2	11.1^bc^ ± 0.6	0.96^b^ ± 0.11	3.7 ± 0.5	214.2 ± 3.2	99.1^a^ ± 4.1	0.260^bc^ ± 0.005
P value	0.99	0.28	0.08	0.18	0.03	0.047	0.32	0.41	0.03	0.03

Means with atleast one common superscript in a column do not differ significantly (P > 0.05).

The CoQ10 supplementation did not affect the dressing per cent, giblet weight, gizzard, liver, spleen weight and intestinal length at various energy levels. Whereas, the weight of heart (per cent of body weight) was found to be significantly lower in low energy diet with 40 mg/kg of CoQ10 supplementation and high energy diet at 20 mg/kg of CoQ10 in diet over the respective unsupplemented groups. The abdominal fat percentage was significantly reduced by the CoQ10 supplementation at 40 mg/ kg in both normal and high energy but not in low energy diet fed birds. CoQ10 supplementation at 20 mg/kg did not affect the abdominal fat percentage. The reduction in the abdominal fat at 40 mg of CoQ10/kg in normal and high energy diet might have been due to the better utilization of energy for muscle growth, however, the performance in terms of body weight gain nor feed efficiency were not significantly better in their groups. The intestinal length (cm) expressed as per kg body weight was significantly lower in normal energy group supplemented with 20 and 40 mg CoQ10/kg of diet but the length was not affected by both the levels of CoQ10 in low and high energy groups. No reason could be assigned for reduction in intestinal length in normal energy diet supplemented with CoQ10. In normal energy, CoQ10 supplementation did not affect the ascites heart index whereas low energy diet with 40 mg/kg CoQ10 supplementation and high energy diet with 20 mg/kg of diet CoQ10 supplementation, ascites heart index was significantly reduced over the unsupplemented groups.

### Cost effectiveness

The feed cost of the different experimental feeds and the cost of feeding per kg of gain of CoQ10 supplementation at different energy levels are presented in Table 7.

**Table 7:** **Influence of Coenzyme Q10 and energy each at three levels on the cost of feed/kg live weight gain**

	NE	LE	HE	NE + 20	LE + 20	HE + 20	NE + 40	LE + 40	HE + 40
Cost of feed/kg (Rs.)									
**Broiler 0–2 weeks diet**	33.22	31.85	34.59	33.31	31.95	34.69	33.41	32.04	34.79
**Broiler 3–4 weeks diet**	33.19	31.65	34.25	33.29	31.74	34.35	33.39	31.84	34.45
**Broiler 5–6 weeks diet**	32.86	31.47	34.11	32.95	31.57	34.20	33.05	31.66	34.30
**0-2 weeks**	312	322	304	287	308	310	304	322	301
**3-4 weeks**	970	940	874	908	897	853	916	840	793
**5-6 weeks**	1930	2184	1931	1949	2134	1931	2013	2117	1974
**Feed cost**	105.98	108.74	106.32	104.01	105.68	106.09	107.27	104.09	105.50
**0-6 week body weight gain**	1948.38	1903.70	2061.63	1931.93	1912.33	2129.27	1910.00	1899.50	1987.27
Feed cost/kg live weight* (Rs.)	54.39^e^ ± 0.15	57.12^i^ ± 0.05	51.57^b^ ± 0.16	53.84^d^ ± 0.10	55.26^g^ ± 0.08	49.83^a^ ± 0.19	56.16^h^ ± 0.09	54.80^f^ ± 0.09	53.09^c^ ± 0.05

Means with atleast one common superscript in a column do not differ significantly (P > 0.05).

The effect CoQ10 supplementation at different energy levels on feed cost per kg live weight gain was significantly low in high energy diet supplemented with CoQ10 at 20 mg/kg (. 49.83) and highest in normal energy supplemented with 40 mg/kg (. 56.16). The general trend was 20 mg/kg supplementation resulted in lower feed cost in all the three levels of energy over the corresponding energy unsupplemented and 40 mg/kg supplemented groups. However, only the low energy group supplemented with 40 mg/kg had lower cost of feeding than the corresponding energy unsupplemented group.

## Discussion

### Growth performance

The non-significance in body weight gain on CoQ10 supplementation were also recorded by Geng et al. (2004a) in broilers, Honda et al. (2010) in growing chickens, Nakamura et al. (1996) using CoQ9 in broilers. However, Geng et al. (2007) reported that the broilers maintained at low environmental temperature (maximum temperature −15°C and minimum - 12°C), had significantly higher weight gain with comparable feed intake and feed efficiency when 40 mg of CoQ10/kg of diet was supplemented. Similarly, Huang et al. (2011) reared birds at 22°C also observed better weight gain between 0 to 21 days of age at 20 or 40 mg/kg of diet CoQ10 supplementation but the growth rate between 21 to 42 days was comparable. Krizman et al. (2012) suggested that continuous supplementation (1 to 42 days) of CoQ10 improved the body weight over the unsupplemented group (where no mention of the rearing temperature was indicated). The studies by Geng et al. (2007) and Huang et al. (2011) were carried out under lower environmental temperature which predisposes pulmonary hypertension syndrome (PHS) which leads to ventricular hypertrophy. This ventricular hypertrophy leads to reduced blood supply to various body tissues; a CoQ10 supplementation significantly reduced the ventricular hypertrophy (Table 6), the higher growth rate may be observed. In the present study, the birds were not under lower environmental temperature, hence no significant difference in growth rate could be observed. The level of CoQ10 did not influence the production score in the present study. However, Nakamura et al. (1996) reported higher production score when CoQ9 was supplemented, where the supplemented CoQ10 reduced the ascites mortality over the control as production score is directly proportional to survival rate. However, in the present study there no mortality of birds due to ascites or any other causes, hence the production score did not vary significantly as compared to Nakamura et al. (1996).

The higher feed intake in low energy diet might be due to the bird’s trying to meet its energy requirement or to meet the critical amino acids lysine, methionine as the diets are formulated to contain the same ratio of energy : lysine; energy : methionine (since low energy diets contain low percentage of lysine and methionine). The feed efficiency was significantly better in high energy than other energy levels which coincide with the observation of Corduk et al. (2007). High energy diets to broilers help maximize energy intake and this higher energy intake maximizes the growth rate (Leeson and Summers 2001). High energy diet fed group had the highest production score than the normal and low energy diet fed groups. The higher production score might be due to higher body weight gain and better feed efficiency because the survival rate was uniform among all the groups.

### Slaughter parameters and ascites heart index

The reduced relative heart weight (g/kg of body weight) was reported even at 20 mg/kg of CoQ10 by Huang et al. (2011) as his study was carried out at minimum temperature range of 19.69 to 22.21°C, the right ventricle to total ventricle (RV/TV) was reduced by CoQ10 supplementation; hence the heart weight had reduced. This might be due to that CoQ10 offer some protection to cardiac myocytes of chicken as explained by Azuma et al. (1985). However, the increase in relative heart weight at 20 mg/kg (Geng et al. 2004a, 2004b and Geng et al. (2007) may be due to better growth performance, predispose to pulmonary hypertension, right ventricular hypertrophy and hence the heart muscle mass might have increased. Similar reduction in AHI due to CoQ10 supplementation was also reported by Azuma et al. (1985), Geng et al. 2004a, 2004b, Geng et al. (2007), Huang et al. (2011). The liver weight was significantly lower in low energy diet group similar to the observation of Cakir and Yalcin (2007) this might be due to enhanced lipogenesis in the liver (Router and Steele 1996; Swennen et al. 2006, Kamran et al. 2008) of birds fed with normal and high energy diet. Ayorinde (1994); Leeson et al. (1996); Raju et al. (2004); Nahashon et al. (2005) found that the per cent of abdominal fat was significantly increased as the dietary energy level increased. More fat deposition, due to the conversion of surplus energy to triglyceride and stored in adipose tissue as stated by Crespo and Gracia (2002). The widening of dietary calorie: protein ratio is also known to increases fat deposition in broilers. In the present study, increased abdominal fat in low energy diet has resulted even when the calorie: protein ratio was same in all treatments. The studies of Mateos et al. (1982) carried out with high fat supplemented ration were reported to reduce the intestinal tract length. This might be due to reduced rate of food passage in the gastro-intestinal tract when compared to birds fed without/less fat.

## Conclusion

There is no significant effect of coenzyme Q10 as such in growth performance, slaughter parameters and cost effectiveness in broilers. The dietary supplementation of CoQ10 at 20 mg/kg in high energy diets leads to better body weight gain, feed efficiency and cost of production which might be an indication of energy – CoQ10 interaction in the body which needs further experiments to explore this.
